# Stanniocalcin-1 Alleviates Contrast-Induced Acute Kidney Injury by Regulating Mitochondrial Quality Control via the Nrf2 Pathway

**DOI:** 10.1155/2020/1898213

**Published:** 2020-04-12

**Authors:** Fei Zhao, Li-Xin Feng, Qian Liu, Hong-Shen Wang, Cheng-Yuan Tang, Wei Cheng, Yin-Hao Deng, Xi Wu, Ping Yan, Xiang-Jie Duan, Jin-Cheng Peng, Shao-Bin Duan

**Affiliations:** Department of Nephrology, The Second Xiangya Hospital, Central South University, Hunan Key Laboratory of Kidney Disease and Blood Purification, Changsha, Hunan 410011, China

## Abstract

Contrast-induced acute kidney injury (CI-AKI) is the third common cause of acute kidney injury (AKI), which is associated with poor short- and long-term outcomes. Currently, effective therapy strategy for CI-AKI remains lacking. Stanniocalcin-1 (STC1) is a conserved glycoprotein with antiapoptosis and anti-inflammatory functions, but the role of STC1 in controlling CI-AKI is unknown. Here, we demonstrated a protective role of STC1 in contrast-induced injury in cultured renal tubular epithelial cells and CI-AKI rat models. Recombinant human STC1 (rhSTC1) regulated mitochondrial quality control, thus suppressing contrast-induced mitochondrial damage, oxidative stress, inflammatory response, and apoptotic injury. Mechanistically, activation of the Nrf2 signaling pathway contributes critically to the renoprotective effect of STC1. Together, this study demonstrates a novel role of STC1 in preventing CI-AKI and reveals Nrf2 as a molecular target of STC1. Therefore, this study provides a promising preventive target for the treatment of CI-AKI.

## 1. Introduction

Acute kidney injury has become a worldwide public health problem [1]. The third most common cause of AKI is contrast medium-induced acute kidney injury which happens to 30% of patients who were administered intravascularly with contrast media [2]. CI-AKI is characterized by a sharp decrease in glomerular filtration rate (GFR) followed by an increase in serum creatinine concentration or oliguria after intravascular administration of contrast media [3, 4]. A large and growing body of researches show that CI-AKI is related to serious short- and long-term adverse outcomes, such as short-term mortality, prolonged hospitalization, increased hospital-related costs, and chronic kidney disease risk [4, 5].

Many pathophysiological processes contribute to the development of CI-AKI, such as renal medullary ischemia, direct tubular cytotoxicity of contrast, overproduction of reactive oxygen species (ROS), mitochondrial damage, and mitophagy [1, 6–9]. Most guidelines suggest that expanding intravascular volume is more reasonable in preventing CI-AKI [10, 11]. A recent study shows that no prophylaxis is noninferior and cost-saving in preventing CI-AKI, compared with a common intravenous hydration prevention strategy [12]. To date, no optional pharmaceutical has been demonstrated to effectively prevent or treat CI-AKI.

STC1, an evolutionarily conserved glycoprotein, is ubiquitously expressed in some tissues, such as the kidney [13]. STC1 is considered to function in an autocrine/paracrine or bloodborne manner [14–16]. Then, it targets the inner mitochondrial membrane, and more than 90% of cellular STC1 immunoreactivity is in mitochondria [17]. Evidences suggest that mammalian STC-1 has various biological effects on the prevention of cell ischemia [18], suppression of inflammatory, and reduction of ROS generation, apoptosis, and necrosis [19]. Moreover, emerging evidences present that STC1 can function as an intracrine factor, targeting and working in the mitochondria [18]. Exogenous STC1 can be internalized quickly by the mitochondria in inflammatory cells [19]. Therefore, STC1 may be a potential therapeutic pathway, for example, through acting directly on the mitochondria of the kidney.

In the present study, CI-AKI animal and cell injury models were used to clarify the protective role of STC1 in contrast-induced damage to renal tubular epithelial cells. Additionally, we explored the molecular mechanisms about whether the STC1-Nrf2 pathway participated in this protective role by regulating the imbalance of mitochondrial quality control. The results of this study present that the STC1-Nrf2 pathway contributes to resisting contrast medium-induced acute kidney injury through downregulating mitochondrial damage, suppressing oxidant stress, and inhibiting inflammation and apoptosis in the kidney.

## 2. Methods

### 2.1. Cell Culture Studies

The human proximal tubular cell lines (HK-2 cells) were treated with rhSTC1 (50 ng/ml or 100 ng/ml) or (and) TBHQ (40 ng/ml) or (and) ML385 (10 *μ*M) at the same time of iohexol (GE Healthcare, Shanghai, China) intervention to illuminate the effects of rhSTC1 on mitochondrial damage, ROS, inflammation, and cell apoptosis. rhSTC1 (Cat. No. 9400-SO-050) was purchased from R&D Systems (Minneapolis, MN, USA). Diluted by using distilled water, rhSTC1 protein was produced at a final concentration of 100 *μ*g/ml. Tert-butylhydroquinone (TBHQ) and ML385 were purchased from Selleck (State of Texas, USA). STC1 siRNA and negative siRNA control were transfected into the HK-2 cells through using the Lipofectamine 2000 reagent (Life Technologies, USA).

### 2.2. Animal Experimental Design

Before our studies, male Sprague-Dawley rats were maintained for 7 days to acclimatize, according to the animal care rules of the Second Xiangya Hospital of Central South University. The rats were divided into four groups: control group (*n* = 6), CI-AKI group (*n* = 6), CI-AKI group receiving a tail vein injection of rhSTC1 (5 *μ*l) (*n* = 6, rhSTC1, 0.5 *μ*g/rat), and CI-AKI group receiving a tail vein injection of rhSTC1 (10 *μ*l) (*n* = 6, rhSTC1, 1 *μ*g/rat). The CI-AKI rat model was built according to our previous article [6]. The rhSTC1 is a human-derived recombinant protein, but its function has been testified in other previous studies [20, 21]. The nonionic low-osmolar contrast medium, iohexol (GE Healthcare, Shanghai, China), was administrated by intravenous injection via the tail vein; furosemide (Shanghai, China) was intramuscularly injected as previously described. In brief, all rats were dehydrated for 48 h, then received furosemide (10 ml/kg) 30 minutes before iohexol (15 ml/kg) injection, and the rhSTC1 was added at the same time of iohexol injection. Twenty-four hours later, CI-AKI rat models were built. The control group did not receive iohexol injection.

### 2.3. Assessment of Renal Function and Histopathology

Ways of measuring blood urea nitrogen (BUN), serum creatinine (Scr), and renal pathology were described in our recent studies [6]. Briefly, kidney tissue was fixed and embedded, and tissue sections (4 *μ*m thick) were then stained with hematoxylin-eosin for histopathological analysis. For semiquantitative analysis of the changes of kidney tissue, 15 high-magnification (×200) fields of the cortex and outer medulla were selected randomly. The specimens were scored as previously described [22]: no injury (0), mild < 25% (1), moderate < 50% (2), severe < 75% (3), and very severe > 75% (4).

### 2.4. dUTP Nick-End Labeling Assay

The terminal deoxynucleotidyl transferase-mediated dUTP nick-end labeling (TUNEL) assay was performed with a commercial kit (In Situ Cell Death Detection Kit; Roche, Basel, Switzerland) to detect DNA strand breaks, according to the manufacturer's protocol. The number of TUNEL-positive and total cells in different kidney sections was counted in five different views per section by using a fluorescence microscope (Motic, BA410E). TUNEL-positive cells were expressed as a percentage of total cells.

### 2.5. Western Blotting

Cells or renal cortical tissues (*n* = 3from each group) were isolated, as described previously [6, 7]. Nuclear and cytoplasm protein were extracted according to the operating instructions. Protein concentration was measured by using the BCA Protein Assay Reagent Kit (Beyotime Institute of Biotechnology, Shanghai, China). Equal amounts of proteins were separated on SDS-polyacrylamide gel before transferred to nitrocellulose membranes, then probed with primary antibodies against cleaved caspase 3, LC3II (1 : 1000, Cell Signaling Technology, Boston, USA), nuclear factor- (erythroid-derived 2-) like 2 (Nrf2), Kelch-like erythroid cell-derived protein with CNC homology- (ECH-) associated protein1 (Keap1), TOMM20 (1 : 1000, Proteintech, Chicago, USA), [NOD]-like pyrin domain containing protein 3 (NLRP3), dynamin-related protein 1 (Drp1), heme oxygenase-1 (HO-1), PTEN-induced putative kinase 1 (PINK1), inflammasome and high mobility group box 1 (HMGB1), mitofusin-2 (mfn2), RBR E3 ubiquitin protein ligase (Parkin), sequestosome 1 (P62) (1 : 1000, Abcam, Cambridge, UK), and *β*-actin (1 : 1000, Proteintech, Chicago, USA); then, horseradish peroxidase-conjugated secondary antibodies (1 : 5000, Proteintech, Chicago, USA) was used as secondary antibody. Tanon 5200 Multi image analysis software (Tanon, Shanghai, China) was used to analyze the results.

### 2.6. Immunohistochemistry Staining

For immunohistochemistry staining, kidney tissues were fixed, deparaffinized, blocked, and reduced nonspecific binding by 0.1% Triton X-100. The slides were then incubated with primary antibodies at 4°C overnight and counteracted with HRP-conjugated secondary antibody for 1 hour at room temperature. The color was developed with a DAB kit (Beijing Zhongshan Jinqiao Biotechnology, ZLI-9018).

### 2.7. Quantification of Mitochondrial DNA Content

Renal tissues were separately collected from the control group, the CI-AKI group, and the rhSTC1 treatment group. mtDNA was extracted and measured as previously described [6]. Plasma DNA from rats of each group was collected by using the QIAamp DNA Mini and Blood Mini Kit (Qiagen, Germantown, MD), and plasma mtDNA were calculated by quantitative PCR through a LightCycler480 System with SYBR Premix Ex Taq II (Takara, Japan). The relative abundances of plasma mtDNA were indicated as threshold cycles (Tc). And higher Tc represents lower levels of mtDNA.

### 2.8. Determination of Reactive Oxygen Species

Mitochondrial ROS in HK-2 cells was evaluated by using MitoSOX Red (Invitrogen Corporation, USA) as previously described [7], according to the manufacturer's instruction. Briefly, cells growing in cell culture plates were rinsed and incubated in MitoSOX (Invitrogen Corporation, USA) for 8 minutes at 37°C in the dark. DAPI (Invitrogen Corporation, USA) was used to label nucleus. Dihydroethidium (DHE) (Thermo Fisher Scientific, D11347) was used to stain ROS in kidney tissues. The kidney was cut and rinsed in cold PBS and then was placed in Tissue-Tek optimal cutting temperature compound, followed by snapping frozen in liquid nitrogen. Twenty *μ*m thick kidney slices isolated freshly were incubated in 10 *μ*M DHE in a humidified/dark chamber at 37°C for half an hour and then counterstained with DAPI (Invitrogen Corporation, USA). The distribution of ROS was measured by a confocal laser scanning microscope LSM780 (Carl Zeiss Jena, German). In order for quantification, fluorescent density within 10 random optical sections was determined with ImageJ software.

### 2.9. Transmission Electron Microscope Analysis (TEM)

HK-2 cells were treated as mentioned above. Then, the cells were collected and undergone dehydration, osmosis, embedding, sectioning, and staining, as previously described [7]. Typical images were captured by a Hitachi H7700 electron microscope.

### 2.10. Immunofluorescence Colocalization Analysis

Mitochondrial morphology and mitophagy were evaluated as previously described [7]. The cells, incubating in MitoTracker Red for 8 min, then were washed, fixed, rinsed, and permeabilized. 1% BSA was added to block the cells for 60 minutes at room temperature, and the cells then were incubated with the primary antibodies anti-LC3II (1 : 100), anti-Drp1 (1 : 100), anti-dsDNA antibody (1 : 100), and anti-Nrf2 (1 : 100) and secondary antibodies conjugated with FITC in vitro. DAPI (Invitrogen Corporation, USA) was stained in the dark. Significant images were measured by a confocal laser scanning microscope LSM780 (Carl Zeiss, Jena, German).

### 2.11. Statistical Analysis

Statistical analysis was conducted by using the GraphPad Prism and SPSS 19.0 software. All quantitative variables were presented as the mean ± standard error of mean (mean ± SE) and were compared by using a paired or unpaired Student's *t*-test or a one-way analysis of variance (ANOVA) followed by the LSD test for post hoc comparisons. A *p* value of <0.05 was considered significant.

## 3. Results

### 3.1. Iohexol Induces Cytotoxicity and Mitochondrial Dysfunction In Vitro

**Figure 1:**
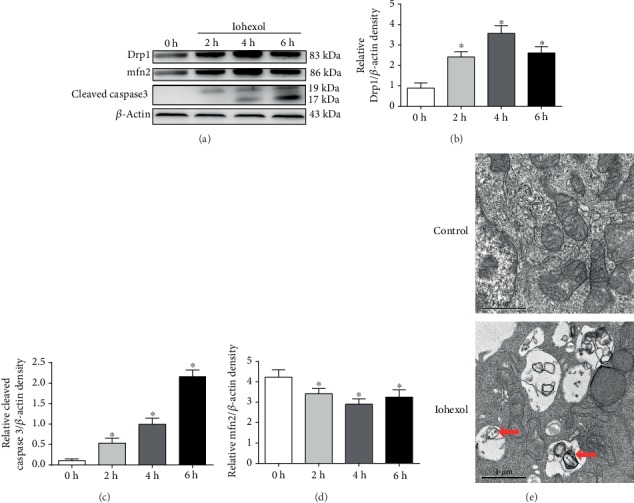
Iohexol induces cytotoxicity and mitochondrial dysfunction in HK-2 cells. HK-2 cells were treated with iohexol (200 mg I/ml) at indicated time points. (a) Drp1, mfn2, and cleaved caspase 3 were measured by Western blot assay (*n* = 3). Iohexol treatment caused a decreased expression of mfn2 and increased expression of Drp1 and cleaved caspase 3. (b–d) Quantification of average Western blot band intensities. (e) Representative images. HK-2 cells were treated with iohexol (200 mg I/ml) for 4 hours and then collected for transmission electron microscope analysis. Mitophagy was labeled with red arrow. Values were presented as mean ± SE. ^∗^*p* < 0.05, compared with the control group.

In order to maintain mitochondrial network balance, mitochondria undergo unceasing fission and fusion processes which are regulated by profission proteins (Drp1), profusion proteins (mfn2), and mitochondrial membrane protein (TOMM20). Dysfunctional mitochondria exhibit fragmentation, increased expression of Drp1, and ROS and decreased expression of mfn2 and TOMM20. As we previously reported, iohexol could induce a decrease in cell viability and an increase in ROS generation [7]. As shown in Figure 1, we further demonstrated that iohexol induced increased expression of Drp1 and cleaved caspase 3 and decreased expression of TOMM20 and mfn2. Additionally, iohexol treatment induced mitochondrial swelling, mitochondrial fragmentation, and mitophagy (Figure 1(e)). These results indicated that iohexol led to mitochondrial damage, mitophagy, and cell apoptosis.

### 3.2. rhSTC1 Treatment Attenuates Inflammation Injury and Mitochondrial ROS Generation in HK-2 Cells

**Figure 2:**
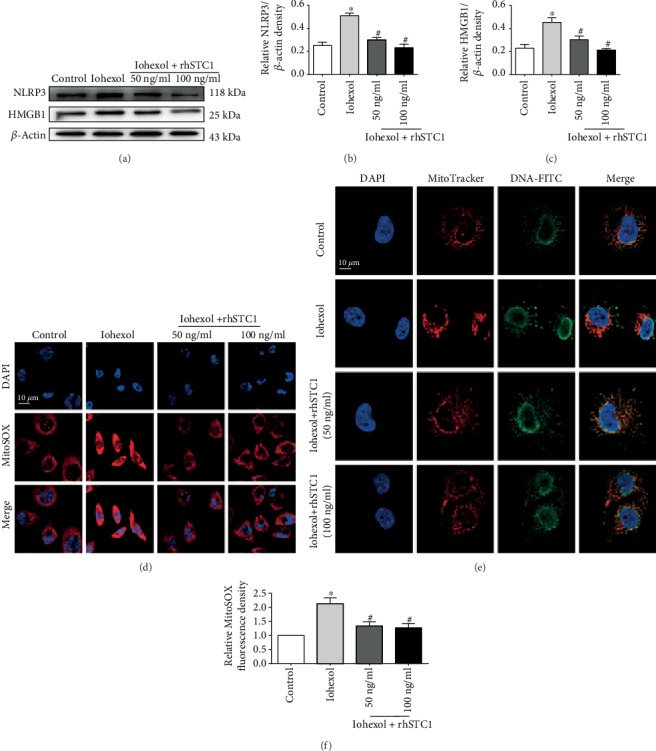
rhSTC1 treatment attenuates inflammation injury and mitochondrial ROS generation in HK-2 cells. HK-2 cells were treated with iohexol (200 mg I/ml) with or without rhSTC1 at different concentrations (50 ng/ml, 100 ng/ml) at indicated time courses, respectively. (a) Western blot analysis of the expression of HMGB1 and NLRP3 (*n* = 3). (b, c) Quantification of average Western blot band intensities. (d–f) Representative images of mitochondrial ROS generation (d) and mitochondrial DNA release (e) (*n* = 3). DAPI staining was performed to label nuclear (blue). Mitochondrial ROS was labeled by MitoSOX (red). Mitochondrial DNA was labeled by the indicated antibody (green). Fluorescence images were taken by a LSM780 confocal microscope (magnification, ×630). Values were presented as mean ± SE. ^∗^*p* < 0.05, compared with the control group. ^#^*p* < 0.05, compared with the iohexol group.

Mitochondrial dysfunction leads to an increase in mitochondrial ROS generation and the release of mitochondrial DNA (mtDNA). Extensive mtDNA lesions exacerbate mitochondrial oxidative stress and act as damage-associated molecular patterns to trigger the inflammatory response [9]. To examine whether exogenous rhSTC1 played a role in anti-inflammation and protected mitochondrial function in the HK-2 cells, rhSTC1 was added into the HK-2 cells as an intervention at the same time of iohexol treatment. As shown in Figure 2, iohexol treatment induced increased expression of NLRP3 and HMGB1 (Figure 2(a)), overgeneration of mitochondrial ROS (Figure 2(d)), and mitochondrial DNA release (Figure 2(e)). Treatment with rhSTC1 could decrease the expression of NLRP3 and HMGB1, generation of mitochondrial ROS, and mitochondrial DNA release. These results demonstrated that treatment with rhSTC1 significantly can suppress iohexol-induced inflammation and mitochondrial ROS generation in HK-2 cells.

### 3.3. rhSTC1 Treatment Reduces Mitochondrial Damage and Mitophagy in HK-2 Cells after Iohexol Exposure

**Figure 3:**
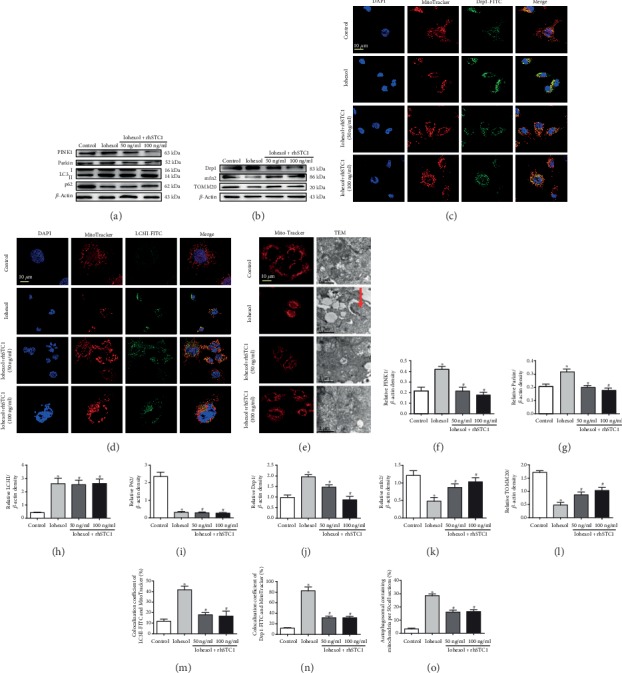
rhSTC1 treatment reduces mitochondrial damage and mitophagy in HK-2 cells after iohexol exposure. (a, b) Western blot analysis of the expression of mitophagy and mitophagy-related protein (*n* = 3). Cells were treated with iohexol (200 mg I/ml) with or without rhSTC1 (50 ng/ml, 100 ng/ml) at an indicated time course, and then the whole cell lysates were collected for Western blot analysis. (c, d) Representative images. HK-2 cells were exposed to iohexol (200 mg iodine/ml) with or without rhSTC1 (50 ng/ml, 100 ng/ml) for 4 hours and then were treated with LC3II-FITC or Drp1-FITC (green) and MitoTracker (red), respectively (*n* = 3). The distribution of LC3II-FITC, Drp1-FITC, and MitoTracker was analyzed by a confocal microscope. Colocalization of LC3II-FITC or Drp1-FITC and MitoTracker was presented as overlapped red and green peaks. (e) Representative images. HK-2 cells were exposed to iohexol (200 mg iodine/ml) with or without rhSTC1 (50 ng/ml, 100 ng/ml) for 4 hours and then were treated with MitoTracker (red) to label mitochondria (*n* = 3) or collected for electron micrograph analysis. Mitophagy was labeled with red arrow. (f–l) Quantification of average Western blot band intensities. (m, n) Semiquantitative fluorescence analysis. (o) Semiquantitative analysis of number of mitophagy. Values were presented as mean ± SE (*n* = 3), and *n* = 3 referred to number of replicates. ^∗^*p* < 0.05, compared with the control group. ^#^*p* < 0.05, compared with the iohexol group.

As reported in our previous studies [7], iohexol could induce mitochondrial damage and mitophagy. The colocalization of LC3II with mitochondria and the expression of P62 are usually used to indicate mitophagy. Mitophagy can be regulated by Drp1, PINK1, and Parkin. Evidences show that PINK1-Parkin-mediated mitophagy plays an important role in AKI [6, 23]. Our results showed that iohexol induced increased expression of Drp1, PINK1, Parkin, and LC3II and decreased expression of mfn2, TOMM20, and P62, which could be reversed by rhSTC1 treatment, except the expression of LC3II and P62 (Figures 3(a) and 3(b)). In addition, the fluorescent confocal of LC3II-FITC or Drp1-FITC with MitoTracker Red was more significantly upregulated, compared with the control group, but rhSTC1 treatment dramatically reversed these increases (Figures 3(c) and 3(d)). We also found that iohexol led to abnormal mitochondrial morphology, mitochondrial fragmentation, and an increase in mitophagy generation, which could also be reversed by rhSTC1 treatment (Figure 3(e)). These results suggested that rhSTC1 treatment could alleviate mitochondrial damage and thus reduced mitophagy. In addition, there was no statistical significance of different concentrations of rhSTC1 functioning on HK-2 cells.

### 3.4. Silencing STC1 Significantly Increases Inflammation Response and Mitochondrial Damage in HK-2 Cells

**Figure 4:**
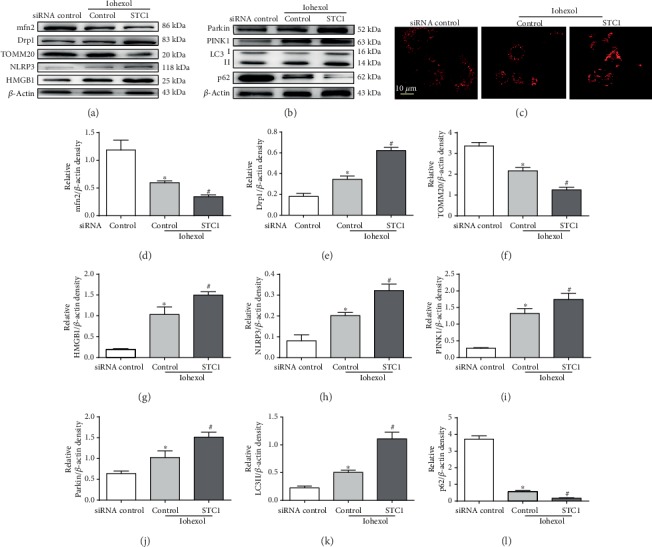
Silencing STC1 significantly increases inflammation response and mitochondrial damage in HK-2 cells. HK-2 cells were firstly transfected with STC1-siRNA, or control siRNA, and 24 hours later, these cells were treated with iohexol (200 mg iodine/ml) for 4 hours with or without rhSTC1 (50 ng/ml). Then, the whole cell lysates were collected for Western blot analysis or fixed for confocal microscopy analysis. (a) Western blot analysis of the expression of inflammation and mitochondrial damage-associated proteins (*n* = 3). (b) Western blot analysis of the expression of mitophagy-associated proteins (*n* = 3). (c) Representative images. HK-2 cells were treated as mentioned above. MitoTracker (red) was used to label mitochondria (*n* = 3). (d–l) Quantification of average Western blot band intensities. Values were presented as mean ± SE. ^∗^*p* < 0.05, compared with the control group. ^#^*p* < 0.05, compared with the iohexol group.

In order to further identify the role of STC1 in anti-inflammation and preventing mitochondrial damage, we observed the changes of inflammation and mitophagy-related proteins. The above study indicated no difference at different concentrations of rhSTC1 in alleviating HK-2 cell injury. Therefore, 50 ng/ml rhSTC1 was used to intervene in HK-2 cells after silencing STC1. Our results showed that iohexol induced increased expression of Drp1, NLRP3, HMGB1, PINK1, Parkin, and LC3II and decreased expression of mfn2, TOMM20, and P62, compared with the control group, which were aggravated by silencing STC1 (Figures 4(a) and 4(b)). We also found that iohexol-induced mitochondrial fragmentation was aggravated by silencing STC1 (Figure 4(c)). In sum, these results indicated the anti-inflammation and protecting mitochondrial role of STC1 on the opposite side.

### 3.5. Antiapoptotic Effects of rhSTC1 in HK-2 Cells

**Figure 5:**
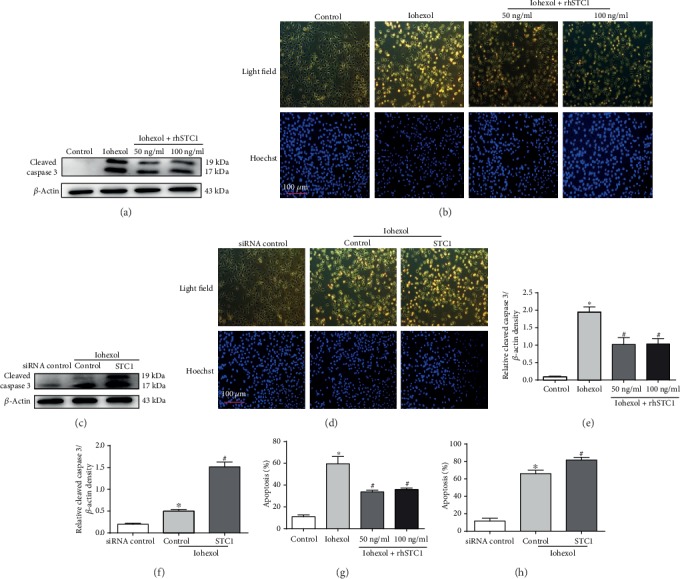
Antiapoptotic effects of rhSTC1 in HK-2 cells. (a, c, and g) HK-2 cells were then exposed to iohexol (200 mg iodine/ml) for 4 hours with or without rhSTC1 at different concentrations (50 ng/ml, 100 ng/ml). (b, d, and h) HK-2 cells were firstly transfected with STC1-siRNA, or control siRNA, and 24 hours later, these cells were treated with iohexol (200 mg iodine/ml) for 4 hours with or without rhSTC1 (50 ng/ml). (a, b) Western blot analysis of cleaved caspase 3 expression in the whole cells (*n* = 3). (c, d) Representative images. Cells were stained with Hoechst 33342. Cellular and nuclear morphology was recorded by phase-contrast and fluorescence microscopy. (e, f) Quantification of average Western blot band intensities. (g, h) The percentage of apoptosis was estimated by counting the cells with typical apoptotic morphology. Values were presented as mean ± SE. ^∗^*p* < 0.05, compared with the control group. ^#^*p* < 0.05, compared with the iohexol group.

To examine the effect of rhSTC1 in preventing cell apoptosis, the expression level of cleaved caspase 3 and apoptosis cells was surveyed by Western blot and fluorescence microscopy. Iohexol induced more significantly increased expression of cleaved caspase 3, compared with the control group, which was alleviated or aggravated by rhSTC1 treatment or silencing STC1, respectively (Figures 5(a) and 5(b)). As shown in Figures 5(c) and 5(d), iohexol markedly led to cell apoptosis, which was further alleviated or aggravated by rhSTC1 treatment or STC1 siRNA. Similar results were obtained from the analysis of apoptosis rate (Figures 5(e)–5(h)). Taken together, these results further indicated that rhSTC1 suppressed iohexol-induced apoptosis in HK-2 cells.

### 3.6. The Protective Effects of rhSTC1 Treatment on Renal Function and Pathological Injury

**Figure 6:**
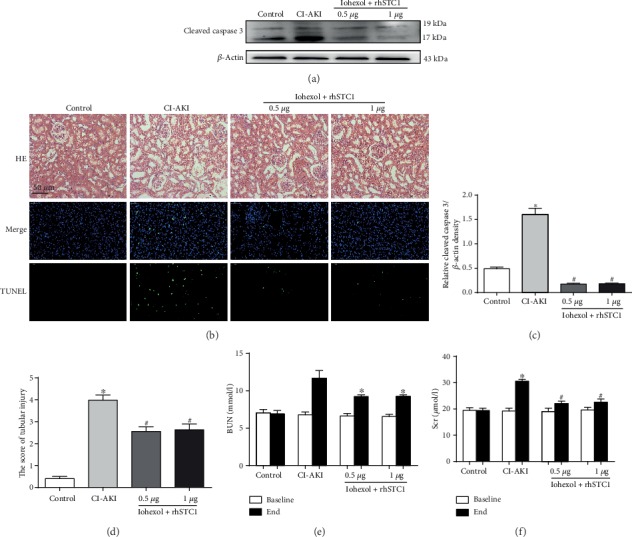
The protective effects of rhSTC1 on renal function and pathological injury in rat kidneys. The way to build CI-AKI rat model was described in our previous article. rhSTC1 at different concentrations (0.5 *μ*g/rat, 1 *μ*g/rat) and iohexol (200 mg iodine/ml) were administrated through the caudal vein at the same time. (a) Western blot analysis of the expression of cleaved caspase 3 in the whole cells (*n* = 3). (b) Representative images of tubular cell injury in rat kidney tissue and immunofluorescent labeling for TUNEL in rat kidney tissue sections of the four groups (×200). Rat kidney tissue was stained for TUNEL (green). Nuclei were stained with DAPI (blue). (c) Quantification of average Western blot band intensities. (d) Quantitative analysis of histologic scoring. (e, f) Changes in the levels of Scr and BUN. Values were presented as mean ± SE. ^∗^*p* < 0.05, compared with the control group. ^#^*p* < 0.05, compared with the CI-AKI group.

We have already constructed a highly effectively novel CI-AKI rat model [6]. As shown in Figures 6(e) and 6(f), Scr and BUN significantly increased after iohexol injection, which were partially restored by rhSTC1 treatment. Kidney histopathological changes were also examined. Iohexol treatment induced more serious detachment and foamy degeneration in tubular cells, which were fewer in the rhSTC1 treatment group (Figures 6(b) and 6(d)). Furthermore, the number of apoptotic cells and expression of cleaved caspase 3 in the iohexol group were significantly higher, compared with the control group. Clearly, rhSTC1 treatment dramatically ameliorated these changes (Figures 6(a) and 6(b)).

### 3.7. rhSTC1 Restores the Expression Level of Drp1 and mfn2 and Reduces ROS Production In Vivo

**Figure 7:**
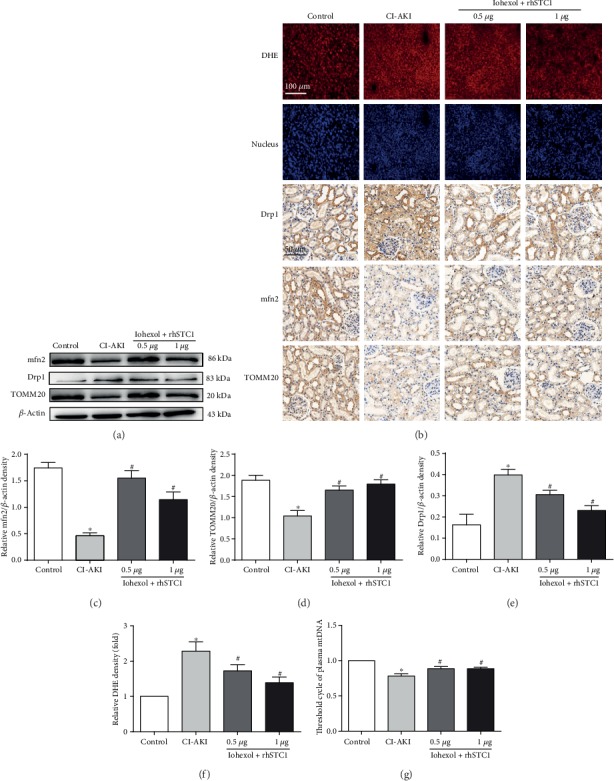
rhSTC1 treatment restores the changes in Drp1 and mfn2 expression and reduces ROS production in the rat kidneys. Rats were treated as mentioned above. (a) Western blot was used to detect the expression of Drp1, mfn2, and TOMM20 (*n* = 3). (b) Kidney sections from the mentioned groups were stained with Drp1, mfn2, and TOMM20 antibodies for IHC analysis (magnification, ×400). Oxidative stress in kidney tubular cells was assessed by using DHE. (c–e) Quantification of average Western blot band intensities. (f) Quantification of tissues stained with DHE. (g) Plasma mitochondrial DNA Tc number decreased after iohexol treatment, which was reversed by rhSTC1. Values were presented as mean ± SE. ^∗^*p* < 0.05, compared with the control group. ^#^*p* < 0.05, compared with the CI-AKI group.

The expression degrees of Drp1, mfn2, and TOMM20 were assessed by using IHC and Western blot. Drp1 expression was upregulated in the tubules of rat kidney, while mfn2 and TOMM20 expression was downregulated, compared with the control group. rhSTC1 treatment presented decreased expression of Drp1 and increased expression of mfn2 and TOMM20, compared with the CI-AKI group (Figure 7(b)). The consistent results were obtained by using Western blot assessment (Figure 7(a)). DHE assays exhibited significant increases in oxidative stress in the CI-AKI rat kidney tubular cells, and rhSTC1 treatment significantly reduced these effects (Figure 7(b)). mtDNA copy numbers decreased in the CI-AKI kidney tissues, and rhSTC1 treatment reversed these changes (Figure 7(g)). All the results suggested that rhSTC1 played a major role in reducing tubular oxidative stress and mitochondrial damage in rat kidney.

### 3.8. Mitophagy-Associated Proteins and Keap1 and Nrf2 Expression Levels Are Regulated by rhSTC1 in Rat Kidney

**Figure 8:**
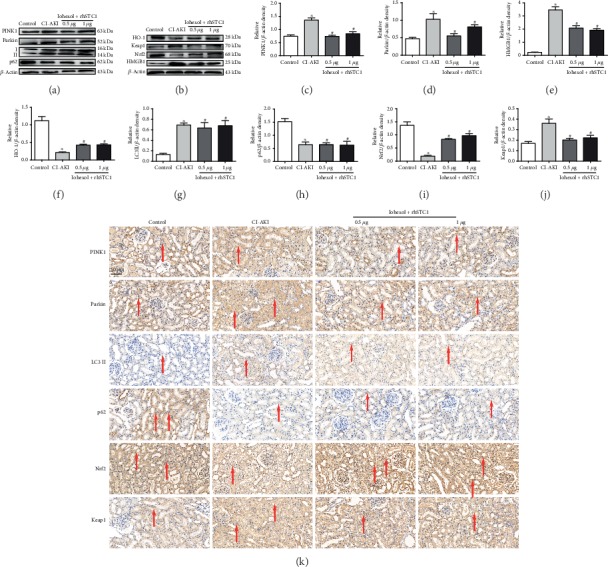
Expression of mitophagy-associated proteins, Keap1 and Nrf2, is regulated by rhSTC1 in rat kidney. Rats were treated as mentioned above. (a, b) Western blot was used to detect the expression of mitophagy-associated proteins, Keap1 and Nrf2 (*n* = 3). (c–j) Quantification of average Western blot band intensities. (k) Kidney sections from the mentioned groups were stained with PINK1, Parkin, LC3II, P62, Nrf2, and Keap1 antibodies for IHC analysis (magnification, ×400). Values were presented as mean ± SE. ^∗^*p* < 0.05, compared with the control group. ^#^*p* < 0.05, compared with the CI-AKI group.

The mitophagy-associated proteins PINK1, Parkin, P62, and LC3II were also evaluated through IHC to identify the potential effect of rhSTC1 on CI-AKI rat tubular cell mitophagy. Increased PINK1, Parkin, and LC3II protein expression levels and decreased expression of P62 were observed in the tubules of CI-AKI rat kidney. All of those changes were reversed by rhSTC1 treatment (Figure 8(k)), except the expression of LC3II and P62. Similar results were obtained concerning PINK1, Parkin, LC3II, and P62, demonstrated by Western blot assays (Figure 8(a)). Notably, Nrf2 and HO-1 expression decreased, and rhSTC1 administration largely restored this change (Figures 8(b) and 8(k)). In contrast, the negative regulator of Nrf2 and Keap1 was upregulated in the CI-AKI rat kidney and significantly decreased by rhSTC1 treatment (Figures 8(b) and 8(k)). These results suggested that rhSTC1 regulated mitochondrial function and ROS generation in CI-AKI kidneys probably via the Nrf2 pathways. And, in vivo, we also revealed that two different dosages of rhSTC1 had similar effects in alleviating contrast medium-induced kidney injury.

### 3.9. rhSTC1 Plays an Anti-Inflammatory, Antiapoptotic, and Mitochondrial Protective Role Partly by Upregulating the Expression of Nrf2

**Figure 9:**
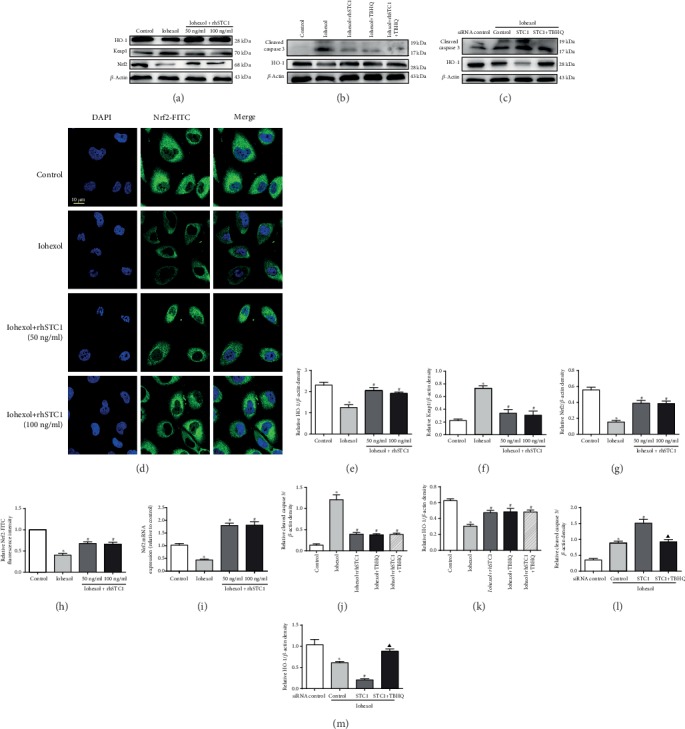
STC1 plays an antiapoptotic role partly by upregulating the expression of Nrf2. (a) HK-2 cells were then exposed to iohexol (200 mg iodine/ml) for 4 hours with or without rhSTC1 at different concentrations (50 ng/ml, 100 ng/ml). Western blot analysis of Nrf2, Keap1, and HO-1 in the whole cells (*n* = 3). (b) HK-2 cells were then exposed to iohexol (200 mg iodine/ml) for 4 hours with or without rhSTC1 (50 ng/ml) or TBHQ (40 ng/ml). Western blot analysis of the expression of cleaved caspase 3 and HO-1 in the whole cells (*n* = 3). (c) HK-2 cells were firstly transfected with STC1-siRNA, or control siRNA, and 24 hours later, these cells were treated with iohexol (200 mg iodine/ml) for 4 hours with or without rhSTC1 (50 ng/ml) or TBHQ (40 ng/ml). Western blot analysis of the expression of cleaved caspase 3 and HO-1 in the whole cells (*n* = 3). (d) Representative images. HK-2 cells were exposed to iohexol (200 mg iodine/ml) for 4 hours and then treated with Nrf2-FITC (green) to label the distribution of Nrf2. Fluorescence images were taken by a LSM780 confocal microscope (magnification, ×630). (e, f, h, j, l, and m) Quantification of average Western blot band intensities. (g) Semiquantitative fluorescence analysis. (i) Expression of STC1 mRNA level. Scale bar, 10 *μ*M. Values were presented as mean ± SE. ^∗^*p* < 0.05, compared with the control group. ^#^*p* < 0.05, compared with the iohexol group. ^▲^*p* < 0.05, compared with the STC1-siRNA treatment group.

**Figure 10:**
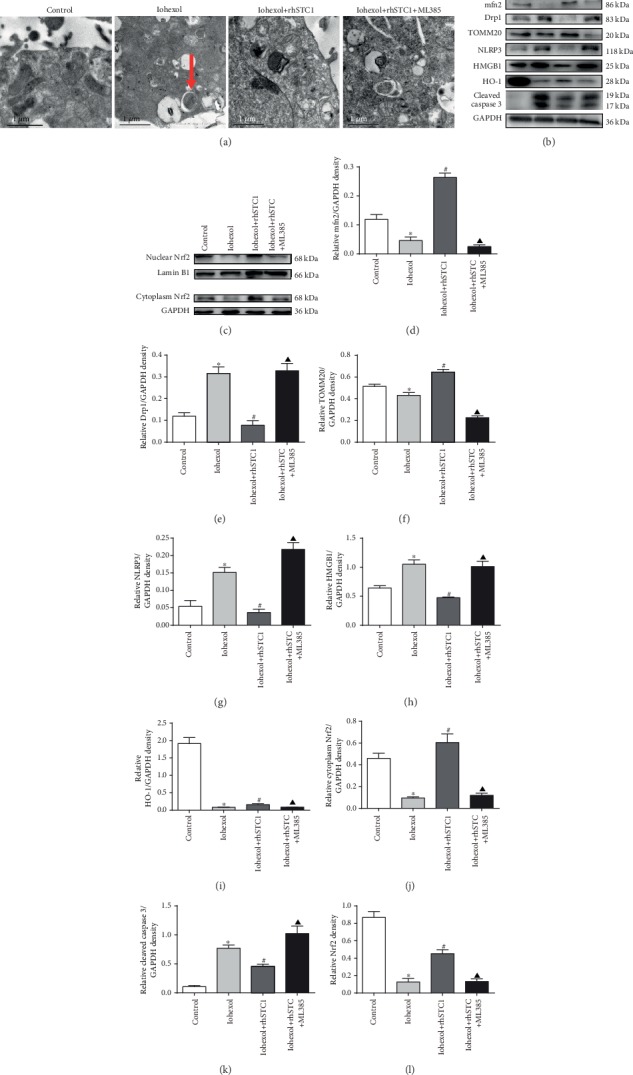
Inhibition of Nrf2 activity partly abolishes the anti-inflammatory, antiapoptotic, and mitochondrial protective role of rhSTC1. HK-2 cells were treated with iohexol (200 mg iodine/ml) for 4 hours with or without rhSTC1 (50 ng/ml) or ML385 (10 *μ*M). (a) Representative images of transmission electron microscope analysis. (b) Western blot analysis of the expression of inflammation, apoptosis, and mitochondrial damage was associated with proteins (*n* = 3). (c) Western blot analysis of the expression of nuclear and cytoplasm Nrf2 (*n* = 3). (d–l) Quantification of average Western blot band intensities. Values were presented as mean ± SE. ^∗^*p* < 0.05, compared with the control group. ^#^*p* < 0.05, compared with the iohexol group. ^▲^*p* < 0.05, compared with the rhSTC1 treatment group.

Although we have illuminated that rhSTC1 treatment ameliorated HK-2 cell mitochondrial damage and apoptosis, the specific mechanism was still unclear. It is also observed that the expression of Nrf2 and Keap1 played an important role in antioxidative response. We assumed that Nrf2 might be a connecting tie between the expression of STC1 and the happening of apoptosis and mitochondrial damage. HO-1, a downstream effector molecule of Nrf2, also has an antioxidant effect. As shown in Figure 9(a), iohexol treatment induced increased expression of Keap1 and decreased expression of Nrf2 and HO-1, which could be reversed by rhSTC1 treatment. Confocal microscopic images also revealed decreased Nrf2 (green) intensity, compared with the control group. These effects were reversed by rhSTC1 treatment (Figure 9(d)). Additionally, rhSTC1 treatment also induced increased expression of Nrf2 mRNA (Figure 9(i)). To further illustrate the relationship between the protective effect of rhSTC1 and the expression of Nrf2, Tert-butylhydroquinone (TBHQ), the inducer of Nrf2 activity [24], was used to observe the expression of cleaved caspase 3 and HO-1 by rhSTC1 treatment or transfection with STC1 siRNA. The results demonstrated that rhSTC1-mediated protection was not abrogated or enhanced by TBHQ treatment, and TBHQ treatment could reverse the increased expression of cleaved caspase 3 and decreased expression of HO-1 induced by STC1 siRNA treatment (Figures 9(b) and 9(c)). A specific Nrf2 inhibitor, ML385, was used to furtherly illuminate the relationship between Nrf2 and STC1. As shown in Figure 10(c), the expression of nuclear and cytoplasm Nrf2 decreased after iohexol treatment, which could be restored by rhSTC1 treatment, and further decreased after rhSTC1 and ML385 intervention. The role of rhSTC1 in anti-inflammation (decreased expression of NLRP3 and HMGB1) and antiapoptosis (decreased expression of cleaved caspase 3) was partly abolished by inhibiting the activity of Nrf2 (Figure 10(b)). Additionally, treatment with rhSTC1 significantly alleviated mitochondrial swelling, fragmentation, vacuoles, and loss of cristae by an electron microscope, which was also partly counteracted by the inhibition of Nrf2 activity (Figure 10(a)). The changes of expression level of mitochondrial damage associated protein (Drp1, mfn2, and TOMM20) further presented that the protective effect of rhSTC1 on mitochondria partly was associated with the activation of Nrf2 (Figure 10(b)). All of these results indicated that rhSTC1 treatment shared some common protective ways with Nrf2 expression. As mentioned, the results demonstrated that rhSTC1 could guard HK-2 cells against contrast medium-induced cytotoxicity partly through the activation of Nrf2.

## 4. Discussion

The pathophysiological effects of STC1 in CI-AKI remain unclear. In this study, we firstly demonstrated the protective role of exogenous rhSTC1 in the contrast-induced cell injury and CI-AKI rat model. Our researches indicated that rhSTC1 treatment dramatically attenuated CI-AKI by reducing mitochondrial damage, reactive oxidative stress, inflammation, and cell apoptosis. We further provided enough evidences and demonstrated that the activation of Nrf2 signaling pathway contributed to the protective role of STC1 in ameliorating the contrast-induced cell injury and CI-AKI rat model. Thus, our results indicated exogenous STC1 had the potential as a novel preventive agent in CI-AKI.

The wide administration of iodinated contrast media in medical practice remains a common cause of AKI and a major reason of iatrogenic nephropathy [25]. The importance of preventing CI-AKI has been demonstrated by emerging studies showing a relation between CI-AKI and mitochondrial quality control. Previous studies suggested that the imbalance of mitochondrial quality control (excessive ROS generation, mitochondrial damage, and mitophagy) played an important role in apoptosis induced by iodinated contrast media [6, 7, 23, 26]. However, the detailed mechanism remains unknown. It is also unclear whether inhibiting mitochondrial ROS generation and alleviating mitochondrial damage can reverse tubular cell damage via regulating mitochondrial quality control.

STC1 is a stress gene, responding to various stimulations, such as hypoxia and cytokines [27, 28]. Some studies have shown a protective role of STC1 in antiglomerular basement membrane glomerulonephritis [19] and LPS-induced lung injury models [21]. Other studies also demonstrated that overexpression of STC1 could protect from hypoxia- or hypercalcemia-induced neuron injury [29]. However, the role of STC1 in CI-AKI is still unknown. Our and other studies showed that mitochondrial damage, overgeneration of mitochondrial ROS, and inflammation played an important role in CI-AKI, and uncontrolled oxidative stress could cause severe tissue damage. In our present study, treatment with rhSTC1 could suppress mitochondrial damage and mitochondrial ROS generation in the kidney in vivo and vitro, suggesting an antioxidative effect of rhSTC1. And inflammation factors, such as NLRP3 and HMBG1, could also decrease caused by rhSTC1 treatment. More importantly, rhSTC1 treatment could lead to a decrease in the expression of cleaved caspase 3 and apoptosis in HK-2 cells and rat kidney. Serum creatinine level and kidney histological changes in CI-AKI were attenuated by rhSTC1 treatment. Taken together, our results firstly suggested that exogenous rhSTC1 attenuated contrast medium-induced acute kidney injury by suppressing the mitochondrial damage, attenuating inflammatory response, and reducing cell apoptosis in the kidney. Moreover, our study further demonstrated that two different concentrations of rhSTC1 showed similar effects on CI-AKI. Therefore, it will be more economical by using fewer dosages of rhSTC1 to treat CI-AKI in the future.

Meanwhile, we found that rhSTC1 treatment caused a decrease in mitochondrial damage and further reduced mitophagy. It seems to contrast to our and other previous results that PINK1-Parkin-mediated mitophagy plays a protective role in CI-AKI [7, 23]. In fact, our present results are consistent with our previous results. First, priming of mitochondria is a determining event to induce autophagic recognition and degradation. In normal pathophysiology, the information of mitochondrial priming for mitophagy is scarce [30]. Once mitochondria exhibit damage and dysfunction, mitophagy is induced to clear damaged or dysfunctional mitochondria for degradation. Our study suggested that rhSTC1 treatment decreased mitochondrial fragmentation and damage demonstrated by confocal and electron microscopy analysis. Second, our results showed that rhSTC1 treatment reduced the expression of PINK1, Parkin, which were important regulator molecules in mitophagy [23, 31]. Third, evidences demonstrated that translocation of Drp1 to mitochondria promoted mitophagy generation. Specific knockdown of Drp1 decreased the mitophagy level [32, 33]. Our results indicated that rhSTC1 treatment decreased the expression of Drp1 and the translocation of Drp1 to mitochondria. Fourth, other researchers found that ROS as a trigger for the generation of PINK1-Parkin mediated mitophagy and the inhibition of ROS generation significantly decreased the expression of Parkin and its translocation to mitochondria [34]. This was also consistent with our results that rhSTC1 treatment decreased ROS generation and then probably decreased mitophagy and mitophagy-related proteins.

We verified the protective effects of rhSTC1 in CI-AKI cell injury and rat models. In order to further explore the possible mechanism, we investigated the effect of the STC1-Nrf2 pathway in contrast medium-induced renal tubular epithelial cell injury. Nrf2 is ubiquitously found in most tissues and acts as an antioxidant and a pivotal factor in regulating oxidative stress and metabolism [35]. Nrf2 is relatively inactive under unstressed conditions, by interacting with an inhibitor protein, Keap1, sequestered in the cytosol [36]. Activation of Nrf2 can inhibit the expression of Drp1 and mitochondrial fission, thus promoting mitochondrial fusion and survival in vivo and in vitro studies [37, 38]. Other studies also show that increased activity of Nrf2 could decrease mitochondrial dysfunction, ROS generation, and apoptosis and is associated with decreased mitophagy level [39, 40]. In our study, iohexol intervention led to decreased expression of nuclear and cytoplasm Nrf2 and increased expression of Keap1, which were reversed by rhSTC1 treatment. In addition, Nrf2 expression was decreased in HK-2 cell exposure to iohexol by confocal microscopic image analysis, compared with the control group. However, both activation of Nrf2 and rhSTC1 treatment had the same effect on alleviating iohexol-induced cytotoxicity, compared with rhSTC1 treatment alone in HK-2 cells. On the other side, STC1 siRNA aggregated HK-2 cell apoptosis, compared to the iohexol intervention group, which was partially alleviated by activation of Nrf2. HMGB1 is a key inflammatory cytokine involved in kidney diseases, and Nrf2 is considered a master regulator of cellular redox signaling, inflammatory pathway. Inhibition of the inflammatory mediator HMGB1 along with upregulation of Nrf2/HO-1 has been demonstrated to significantly improve hypoxia-induced cell injury [41, 42]. Another study also shows that increased Nrf2 activity can attenuate hyperoxia-induced inflammatory by inhibiting the accumulation of extracellular HMGB1 [43]. Additionally, the protective roles of STC1 in preventing mitochondrial damage, apoptosis, and inflammation response were partly abolished by specific inhibition of Nrf2 activity, demonstrated by Western blot and electron microscope analysis. All the data suggested that the STC1-Nrf2 pathway played a protective role in contrast medium-induced renal tubular epithelial cell injury by regulating mitochondrial quality control imbalance.

In conclusion, our researches found a novel beneficial role of STC1 on renal damage in CI-AKI by using in vitro and in vivo models. Treatment with rhSTC1 significantly regulated mitochondrial quality control by decreasing the contrast medium-induced mitochondrial damage, oxidative stress, inflammation, and cell apoptotic injury in CI-AKI. Its protective role was partly attributed to the upregulation of Nrf2 expression and reduction in ROS production. Our finding identifies STC1 as a regulator of Nrf2 in the kidney and brings new insight into the role of STC1 in renal physiology, suggesting that exogenous STC1 has the potential as a novel preventive agent in CI-AKI.

## Data Availability

The data used to support the results of this study are included in the article. The related materials are available from the corresponding author upon reasonable request.

## Abbreviations

AKI: Acute kidney injury
CI-AKI: Contrast-induced acute kidney injury
rhSTC1: Recombinant human stanniocalcin-1
ROS: Reactive oxygen species
Nrf2: Nuclear factor- (erythroid-derived 2-) like 2
Keap1: Kelch-like erythroid cell-derived protein with CNC homology- (ECH-) associated protein1
GFR: Glomerular filtration rate
CKD: Chronic kidney disease
HK-2 cells: Human proximal tubular epithelial cell line
Drp1: Dynamin-related protein 1
mfn2: Mitofusin-2
TOMM20: Translocase of outer mitochondrial membrane 20 homolog (yeast)
mtDNA: Mitochondrial DNA
HMGB1: High-mobility group box-1
NLRP3: NOD-like receptor, pyrin domain containing-3
P62: Sequestosome 1
siRNA: Small interfering RNA
HO-1: Heme oxygenase-1
TBHQ: Tert-butylhydroquinone
DHE: Dihydroethidium
IHC: Immunohistochemistry
PINK1: PTEN-induced putative kinase 1
Parkin: RBR E3 ubiquitin protein ligase
TUNEL: Terminal deoxynucleotidyl transferase-mediated dUTP nick-end labeling
BUN: Blood urea nitrogen
Scr: Serum creatinine.
